# Dimensional Stability of Printed Dental Model: Stability over Time at Different Storage Temperatures

**DOI:** 10.3390/dj14070454

**Published:** 2026-07-20

**Authors:** Yaniv Skvirsky, Tahel Oguen Alon, Mohammad Shehada, Yaniv Mayer, Zvi Gutmacher

**Affiliations:** 1 Department of Prosthodontics, School of Graduate Dentistry, Rambam Health Care Campus (RHCC), Haifa 3525408, Israel; t_oguenalon@rmc.gov.il (T.O.A.); mohammad-s@campus.technion.ac.il (M.S.); zvi@gutmacher.co.il (Z.G.); 2 Department of Periodontology, School of Graduate Dentistry, Rambam Health Care Campus (RHCC), Haifa 3525408, Israel; yaniv.mayer@technion.ac.il

**Keywords:** 3D printing, dental models, storage temperature, dimensional stability, dental resins, accuracy, temperature, additive manufacturing

## Abstract

**Background and Objectives**: Dental models are essential for diagnosis and prosthetic treatment planning and have traditionally been fabricated from gypsum. With the growing adoption of digital workflows, 3D-printed dental models are increasingly used; however, concerns remain regarding their dimensional stability over time. This study evaluated the temporal dimensional changes of gypsum and 3D-printed dental models under different storage conditions and assessed the uniformity of these changes across the models. **Methods**: A fully dentate maxillary typodont was scanned to create a reference model. Sixteen gypsum casts (Type IV dental stone) and 48 3D-printed models from three materials were fabricated. Models were stored at either 4 °C or room temperature and rescanned at predefined intervals over 28 days. Dimensional accuracy was analyzed using 3D inspection software (Geomagic Control X 2022.1.0), with trueness measured by root mean square deviation and precision by standard deviation across the full arch and five predefined regions. Statistical analysis was performed using a Bayesian hierarchical linear model, with significance set at pd > 97.5%. **Results:** Printed models demonstrated significantly higher precision than gypsum models at baseline and day 28 under both storage conditions, with no significant differences among printed materials. In terms of trueness, selected printed groups showed lower deviations than gypsum at baseline, while one material exhibited the best performance at day 28. Gypsum models remained relatively stable over time, whereas printed models showed time-dependent, region-specific dimensional changes, primarily in posterior regions, with maximum deviations of up to 0.026 mm. **Conclusions**: 3D-printed dental models exhibit limited but nonuniform dimensional changes over time, while gypsum models remain comparatively stable. Localized distortions, particularly in posterior regions, should be considered when printed models are used for precise prosthetic applications.

## 1. Introduction

Dental impressions and casts are essential tools in clinical practice. They are frequently used in the diagnosis of dental conditions, in treatment planning and for the fabrication of dental prostheses and appliances. Obtaining an accurate and stable cast model is important as it allows correct diagnosis and fabrication of well-fitting and properly constructed dental devices. Accurate casts mimic patient’s actual anatomical structures and enable proper articulation of maxillary and mandibular arches for occlusal analysis [1].

Three-dimensional printing technology was first introduced in 1986. Since then, a variety of additive manufacturing techniques have been developed. In dentistry, the most widely used are direct light processing (DLP), stereolithography (SLA) and liquid crystal display (LCD) [2]. All these methods are based on vat polymerization, in which a liquid photopolymer resin is selectively light cured [2,3]. Ellakany found that 3D-printed casts had the lowest error rate relative to a reference cast and were comparable to conventional stone casts, with all errors within the clinically acceptable threshold of <0.5 mm [4]. Of these, DLP technology is considered highly accurate, hence its widespread use in dentistry [5]. A systematic review confirmed that most 3D printed models are deemed clinically acceptable, with DLP printers producing models with <100 μm accuracy in most cases [6]. Moreover, printed models exhibit higher impact strength, making them more resistant to fracture [7].

Despite its advantages, several factors may influence the dimensional stability of 3D-printed models. Temperature has been identified as a key factor influencing the dimensional stability of 3D-printed models, primarily due to its effect on polymer behavior; increased temperatures can enhance polymer chain mobility and promote continued polymerization or stress relaxation processes, potentially leading to additional shrinkage or deformation over time [8,9,10]. In one such study it was demonstrated that DLP-printed casts stored at 4 °C exhibited the greatest overall dimensional stability, retaining 99.82–99.86% of original volume in posterior regions after 28 days [11]. The anterior and posterior regions of dental models respond differently to temperature variations. Anterior teeth regions showed less dimensional change at −20 °C (99.42% remaining volume), while posterior regions were most stable at 4 °C [11]. This regional variation is clinically important when considering the intended use of the model [11].

Furthermore, studies have demonstrated that dimensional changes also occur over time [12,13]. One such study found continuous changes (3.3 ± 1.3 µm) over up to 4 weeks [12]. In yet another study contractions of 0.16–0.26 mm. over 21 weeks were reported, with most shrinkage occurring in the first 7 weeks [13].

Another factor that significantly affects the dimensional accuracy and precision of DLP-printed dental models is the type of resin used for the 3D printing model. A comprehensive study comparing four resin brands found significant differences in both trueness and precision at all layer thicknesses [14]. When comparing commercial resins using DLP technology, an experimental low-shrinkage resin demonstrated significantly lower shrinkage and higher accuracy than Die & Model Tan (SprintRay), Formlabs Grey and LCD Grey (Roxel 3D) [15].

3D-printed models using various technologies show mixed results compared to plaster casts. One study found that 3D-printed models showed the lowest error rate relative to the reference cast and were similar to conventional stone casts [4]. Another study demonstrated that models printed using DLP technology exhibited better accuracy than conventional plaster casts, with accuracy values of 46 micrometres compared to 68 micrometres [16]. However, a systematic review found that the accuracy of printed models varies widely, ranging from less than 100 micrometres to more than 500 micrometres, with most models considered clinically acceptable [6].

Despite the growing body of literature, a clear consensus has not yet been established regarding the dimensional stability over time of 3D-printed dental models, particularly under varying storage conditions. Notably, only a limited number of studies have simultaneously evaluated the combined effects of storage duration and temperature, and existing findings are often heterogeneous. Furthermore, there is a lack of data obtained under clinically relevant conditions, which limits the ability to translate current evidence into standardized clinical guidelines [11,13,17,18].

Thus, the aim of the present study is to evaluate the dimensional stability of different 3D-printing materials over time and under different storage conditions and compare it to standard gypsum.

The research hypothesis is that in printed models, there is greater distortion than that observed in gypsum models which is sustainable over time under room temperature and refrigerated storage conditions.

## 2. Materials and Methods

A typodont model (PRO2001-UL-HD-FEM-32; Nissin, Kameoka, Japan) representing a fully dentate maxillary arch was scanned using a laboratory scanner (Identica Blue; Medit, Seoul, South Korea) to serve as the master model.

The typodont model was duplicated 16 times using an irreversible hydrocolloid impression (Hydrogum 5; Zhermack, Badia Polesine, Italy) and poured with Type IV dental stone (Fujirock EP; GC, Tokyo, Japan). Eight gypsum (Group G) casts were stored under refrigeration at 4 °C and eight casts were stored at room temperature at 20 °C. All casts were scanned 1.5 h after pouring (Day 0).

The printed models were fabricated from a single standardized STL file generated from the initial laboratory scan of the typodont, thereby ensuring that all specimens shared an identical reference geometry. The STL file was imported into the printer preparation software and positioned in a horizontal orientation [19,20] on the build platform. All models were manufactured using a Shining 3D AccuFab-CEL printer (Shining 3D, Hangzhou, China) with a layer thickness of 100 µm. Following printing, the models were carefully removed from the build platform and subjected to ultrasonic cleaning in isopropyl alcohol (IPA) for 60 s to eliminate residual uncured resin from the specimen surfaces. After cleaning, the models were allowed to dry completely and were subsequently post-cured in a FabCure 2 curing unit (Shining 3D, Hangzhou, China) for 7 min, in accordance with the manufacturer’s recommended processing protocol. The same printing orientation, layer thickness, cleaning procedure, and post-curing parameters were used for all specimens to minimize manufacturing-related variability. The sample size of eight specimens per group for each storage condition was selected in accordance with established conventions in comparable in vitro studies assessing the dimensional accuracy and stability of 3D-printed dental casts, which typically employed group sizes of eight to ten specimens [11,13,21,22]. A total of 16 models from each of three printing materials were manufactured: Dental Model Resin DM12 Shining (Shining 3D, Hangzhou, China; Group SH), Varseo Wax Model Gray (BEGO, Bremen, Germany; Group B), and KeyModel Ultra Resin, Ivory (Keystone Industries, Gibbstown, NJ, USA; Group K). All models were scanned using a single IOS device (TRIOS3; 3Shape A/S, Copenhagen, Denmark) according to the manufacturer’s recommended protocol immediately after printing (Day −1), after post-processing (Day 0), and subsequently stored under two temperature conditions: eight models at 4 °C and eight models at room temperature (20 °C).

All gypsum and printed models were scanned at predetermined time points: 1, 2, 3, 4, 7, 14, 21, and 28 days. Prior to scanning, refrigerated models were removed from cold storage for approximately one hour to allow thermal equilibration.

**Figure 1:**
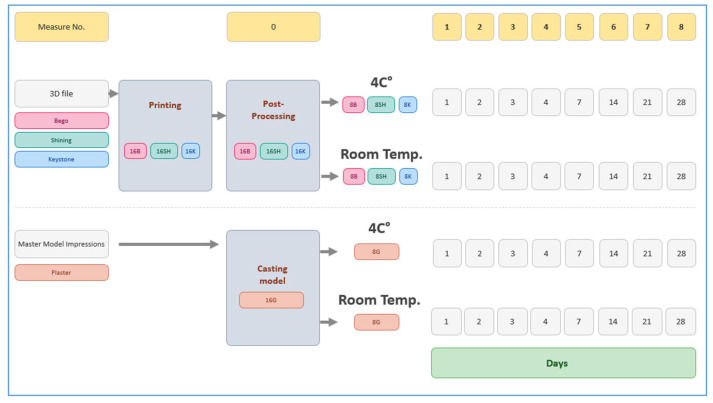
Summary of experiment workflow.

All scans were performed under identical environmental conditions, including room, temperature, and ambient illumination (1000 lux). The scanner was calibrated before use. Two operators (Y.S and T.OA) with a minimum of four years of experience with IOS conducted all scans. To minimize operator fatigue, a one-hour break was taken after every ten scans. The experimental workflow is summarized in Figure 1.

All STL files were imported into Geomagic Control X (v2022.1.0; 3D Systems, Rock Hill, SC, USA) for 3-dimensional analysis. Each IOS dataset was aligned to the reference scan using a best-fit surface-matching algorithm following soft-tissue trimming to reduce gingiva-related variability and allow for more consistent crown-based matching.

Accuracy was calculated as RMS average of the whole model and at five standardized regions: right posterior (second and first right molars), right middle (premolars and canine), midline (lateral and central incisors), left middle (premolars and canine) and left posterior (second and first left molars). The root mean square (RMS) average deviation between test and reference datasets was calculated for each region to represent trueness, whereas standard deviation values represented precision.

Statistical analysis: To properly account for the repeated-measures and nested structure, a hierarchical linear modeling (HLM) framework was adopted. Initial attempts using frequentist linear mixed-effects models encountered convergence problems (e.g., non-convergence and singular fits), likely due to the complexity of the random-effects structure combined with unbalanced data. To address these issues and obtain stable estimation, a Bayesian hierarchical linear model (BHLM) was implemented using the brms (V2.23.0) package in R (V4.4.2).

The model included fixed effects for standardized time, anatomical tooth region, material type, temperature condition, and all interaction terms among these factors. Random effects were specified at the individual level and included a random intercept and random slopes for time, tooth region, and their interaction. A lognormal likelihood was used because the dependent variable was strictly positive and right-skewed, and it demonstrated substantially better predictive performance than the Gaussian alternative based on leave-one-out cross-validation.

Weakly informative priors were specified as follows: Student-t (3, 0.25, 0.10) for the intercept, normal (0, 0.2) for the fixed-effect coefficients, Student-t (3, 0, 0.3) for the residual and random-effect standard deviations, and LKJ (2) for the random-effect correlation structure.

The model was fitted using four chains with 1000 post-warm-up iterations per chain, yielding 4000 posterior draws. Across 1633 monitored parameters, convergence diagnostics indicated good mixing, with a mean R-hat of 1.002 (SD = 0.002; R-hat was calculated as a split chain diagnostic comparing within chain and between chain variation, with values near 1 indicating convergence), a mean bulk effective sample size of 2338 (SD = 1075), and a mean tail effective sample size of 2677 (SD = 698).

To quantify the strength and direction of effects, the probability of direction (pd) was reported. The probability of direction is defined as the proportion of the posterior distribution that lies above zero (for positive effects) or below zero (for negative effects). It can be interpreted as the certainty that an effect is strictly positive or strictly negative, given the model and data.

For readers accustomed to frequentist inference, pd with two-sided *p*-values [23] via:

p=2(1−pd)

Approximately:

• A two-sided *p*-value of 0.10 corresponds to pd ≈ 0.95;
• A two-sided *p*-value of 0.05 corresponds to pd ≈ 0.975;
• A two-sided *p*-value of 0.01 corresponds to pd ≈ 0.995;
• A two-sided *p*-value of 0.001 corresponds to pd ≈ 0.9995.

Statistical significance was set at pd > 97.5%.

## 3. Results

**Figure 2:**
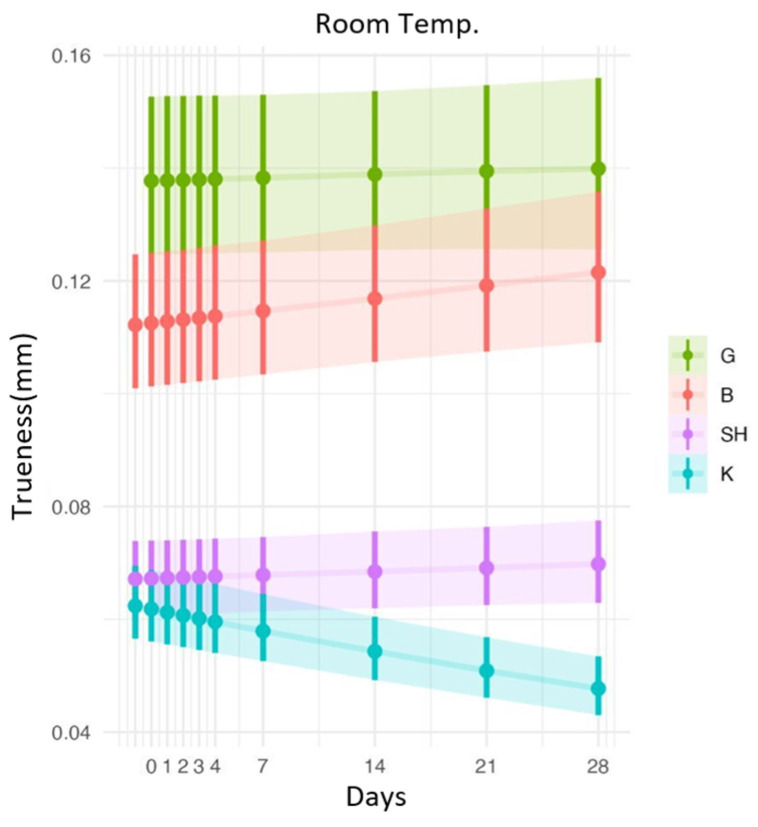
Trueness deviation of gypsum (G) and printed model groups, Shining (SH), Bego (B), and Keystone (K), from day 0 to day 28 under room temperature conditions. Trueness deviation is expressed as the root mean square deviation between the scanned model and the reference digital model, in millimeters. Lower values indicate greater trueness. Each color represents one material group. Points and lines represent the posterior median of the model predicted trueness deviation at each time point. Vertical bars and shaded bands represent the 95% posterior credible intervals.

When comparing models stored at room temperature (Figure 2) at day 0, precision in groups SH, B, and K (0.005, 0.007, 0.014 mm respectively) was significantly superior to in group G (0.046 mm, pd > 0.975), with no differences between the other groups (pd < 0.975). Comparing trueness, SH (0.068 mm), and K (0.069 mm), were significantly (pd > 0.975) better than Groups B and G (0.108 mm and 0.148 mm respectively).

On day 28, precision values (0.007, 0.010 and 0.010 mm) remained significantly better in SH, K, and B compared with G (0.044 mm, pd > 0.975), with no differences between SH, K, and B (pd < 0.975). For trueness at day 28 group K showed the best value (0.056 mm), followed by SH (0.068 mm), while group B (0.116 mm) and G (0.152 mm) showed relatively high deviation.

**Figure 3:**
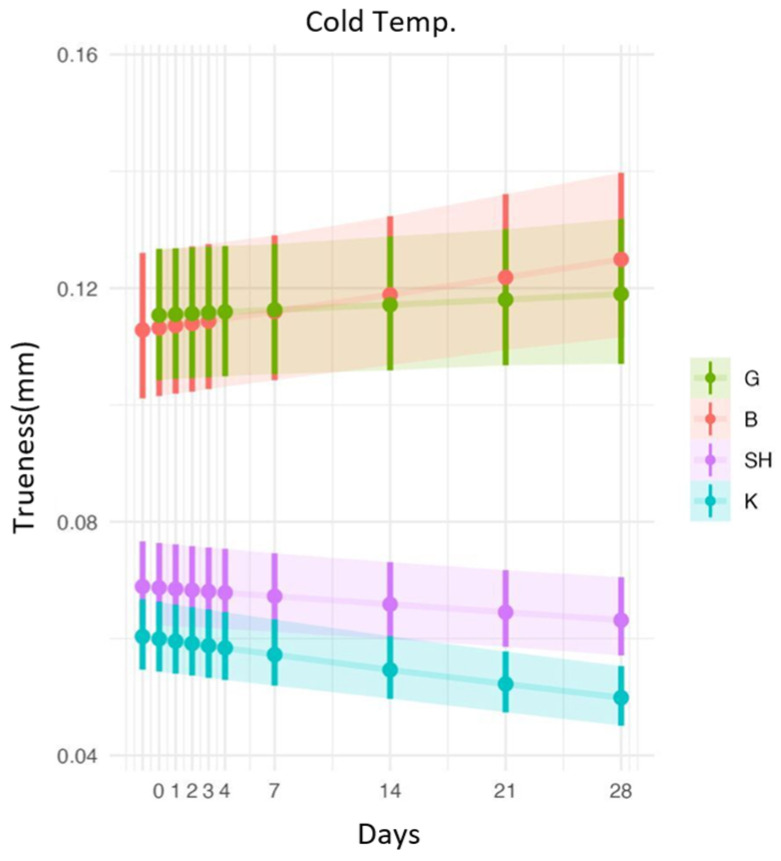
Trueness deviation of gypsum (G) and printed model groups, Shining (SH), Bego (B), and Keystone (K), from day 0 to day 28 under cold storage conditions. Trueness deviation is expressed as the root mean square deviation between the scanned model and the reference digital model, in millimeters. Lower values indicate greater trueness. Each color represents one material group. Points and lines represent the posterior median of the model predicted trueness deviation at each time point. Vertical bars and shaded bands represent the 95% posterior credible intervals.

For cold-stored models (Figure 3), at day 0, precision in SH, B, and K (0.005, 0.005, 0.007 mm) was significantly superior to G (0.033 mm, pd > 0.975), with no differences among SH, K, and B (pd < 0.975). Group K showed the lowest trueness (0.063 mm), followed by SH (0.070 mm), whereas B (0.115 mm) and G (0.124 mm) demonstrated higher values. Significant differences were observed among groups, except between B and G (pd > 0.975).

On day 28, precision values (0.007, 0.010 and 0.006 mm) remained significantly better in SH, B, and K compared with G (0.044 mm, pd > 0.975), with no differences between SH, K, and B (pd < 0.975). For trueness at day 28 group K showed the best value (0.055 mm), followed by SH (0.064 mm) while groups G (0.127 mm) and B (0.128 mm) showed relatively high deviation.

**Figure 4:**
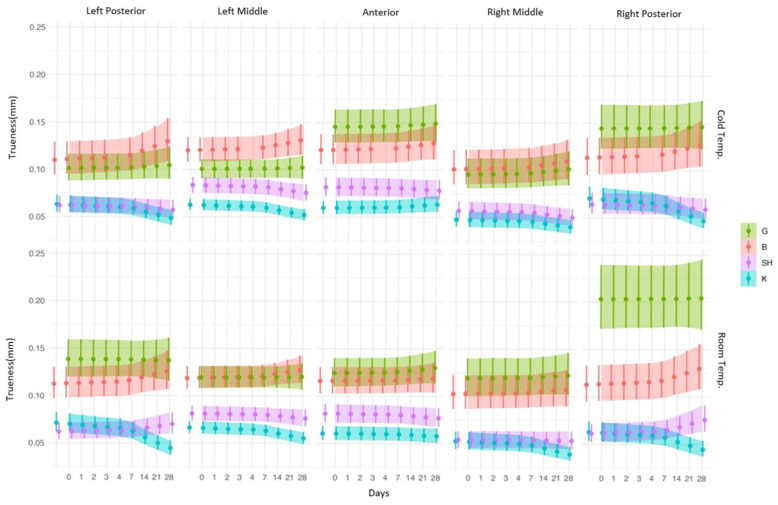
Regional trueness deviation of gypsum (G) and printed model groups, Shining (SH), Bego (B), and Keystone (K), from day 0 to day 28 under room temperature and cold storage conditions. Trueness deviation is expressed as the root mean square deviation between the scanned model and the reference digital model, in millimeters. Lower values indicate greater trueness. Results are shown separately for the anterior region (A), left middle region (LM), right middle region (RM), left posterior region (LP), and right posterior region (RP). Each color represents one material group. Points and lines represent the posterior median of the model predicted regional trueness deviation at each time point. Vertical bars and shaded bands represent the 95% posterior credible intervals.

We then explored trueness in various areas in the models. (Figure 4; Table 1). On day 0, group G demonstrated nonuniform trueness across regions in the two groups, which reflected both impression method and gypsum setting. In room temperature group, the highest deviation was observed in the right posterior region (RP: 0.227 mm), which was significantly greater than in RM (0.146 mm), A (0.133 mm), LM (0.121 mm), and LP (0.143 mm) (pd > 0.975). Similarly, in cold storage group, higher trueness values were recorded in the anterior (A: 0.155 mm) and right posterior (RP: 0.160 mm) regions compared with RM (0.104 mm), LM (0.098 mm), and LP (0.097 mm) (pd > 0.975). In contrast to the printed models, group G demonstrated a change primarily in the spatial distribution of trueness rather than in magnitude, with negligible changes over time from day 0 to day 28 under both storage conditions.

For the printed models at room temperature, posterior regions demonstrated the most notable changes (day 0 vs. 28). Group SH showed small but significant changes (LP: 0.058 vs. 0.066 mm; RP: 0.056 vs. 0.064 mm, pd > 0.975), with minimal changes in anterior and middle regions. Group B demonstrated overall changes in trueness values, most evident posteriorly (RP: 0.108 vs. 0.125 mm, pd > 0.975; LP: 0.106 vs. 0.123 mm, pd = 0.961). Group K showed marked changes over time, particularly in posterior and middle regions (LP: 0.080 vs. 0.054 mm; LM: 0.080 vs. 0.063 mm; RM: 0.054 vs. 0.043 mm; RP: 0.076 vs. 0.057 mm, pd > 0.975), while anterior values remained similar (0.061 vs. 0.063 mm).

Under cold storage conditions, temporal changes remained region-dependent and were most prominent posteriorly. Group B exhibited changes across all regions, with the largest differences in posterior areas (LP: 0.118 vs. 0.130 mm; RP: 0.117 vs. 0.130 mm, pd > 0.975), and smaller, near-significant differences in LM (0.118 vs. 0.132 mm, pd = 0.94) and RM (0.101 vs. 0.114 mm, pd > 0.93). Group SH showed slight changes at day 28, with significant differences in middle regions (LM: 0.082 vs. 0.076 mm; RM: 0.055 vs. 0.050 mm, pd > 0.975). Notably, group K demonstrated the most pronounced changes, with significant differences in posterior (LP: 0.071 vs. 0.053 mm; RP: 0.076 vs. 0.052 mm, pd > 0.975) and middle regions (LM: 0.067 vs. 0.057 mm; RM: 0.049 vs. 0.043 mm, pd > 0.975), while anterior values remained relatively unchanged (0.064 vs. 0.061 mm, pd > 0.94).

When comparing the magnitude of changes (Δ) between day 0 and day 28, similar ranges were observed under both storage conditions, with no consistent evidence for greater changes under cold storage. At room temperature, posterior regions demonstrated changes of approximately 0.008–0.017 mm in groups SH and B, and up to 0.026 mm in group K (LP: 0.080 vs. 0.054 mm). Under cold storage, posterior changes were of comparable magnitude, reaching approximately 0.012–0.013 mm in group B (LP: 0.118 vs. 0.130 mm; RP: 0.117 vs. 0.130 mm) and up to 0.024 mm in group K (RP: 0.076 vs. 0.052 mm). Changes in middle regions were generally smaller but followed a similar pattern across conditions.

## 4. Discussion

The results partially support the research hypothesis. Printed models showed dimensional changes over time. Gypsum models remained more stable overall, despite regional variability.

Significant differences were observed among the tested printed materials. This indicates that resin type plays a critical role in both trueness and precision. Group K showed the best trueness values at both time points and under both storage conditions. Group SH followed, while group B showed the greatest deviations. These findings agree with previous studies reporting that resin composition significantly affects dimensional accuracy and stability [14,15]. Bor et al. [14] evaluated orthodontic models printed with an LCD printer. They compared several commercial resins across multiple printing designs and layer thicknesses. Significant differences in trueness and precision were found between materials, and certain resins consistently outperformed others. Similarly, Ling et al. [15] compared a newly developed experimental resin with commercially available materials. They assessed volumetric shrinkage, accuracy, and mechanical properties. Lower shrinkage was associated with significantly higher accuracy. Variations in polymerization behavior, shrinkage characteristics, and internal stress relaxation may explain these discrepancies. In particular, volumetric shrinkage and post-curing processes influence long-term dimensional stability. Some resins show improved accuracy due to reduced polymerization shrinkage [15].

The gypsum group behaved differently from the printed models. Notably, gypsum casts differed between the room-temperature and cold-storage groups already at day 0, despite identical fabrication protocols. This variation is likely related to the technique sensitivity of gypsum pouring and setting. The casts were fabricated on different days, although all procedures followed the manufacturer’s instructions. Spatial variability in trueness was observed across regions. However, the magnitude of change over time remained minimal under both storage conditions. This suggests that gypsum models are not uniformly accurate across all regions, but they maintain consistent dimensional stability over time. These results agree with previous studies of gypsum behavior. Michalakis et al. [23] evaluated linear dimensional changes of Type IV and V dental stones under different storage conditions over three weeks. They showed that initial expansion peaks within the first 24–96 h. This is followed by contraction and eventual stabilization, with no significant changes after the second week. Similarly, Furuse et al. [24] assessed hygroscopic expansion across multiple gypsum types. Most materials showed controlled and predictable expansion within accepted standards. This emphasizes their reliability in clinical applications. Printed models are subject to ongoing polymer-related changes [15]. In contrast, gypsum casts undergo a well-characterized setting and maturation process. As a result, their dimensions remain relatively stable after the initial phase.

A notable finding of this study is the nonuniform distribution of dimensional changes across model regions. Posterior areas showed greater deviations than anterior regions. This pattern was consistent across all printed materials and under both storage conditions. Similar regional variability has been reported before. Lee et al. [11] evaluated the effect of storage temperature on DLP-printed casts. Dimensional changes were assessed in anterior and posterior regions over 28 days. The regions responded differently to environmental conditions, and posterior regions were more susceptible to dimensional changes. The increased distortion in posterior regions in the present study may be attributed to geometric complexity, greater mass, and accumulation of polymerization stresses in these areas.

Temperature did not show a consistent effect on the magnitude of dimensional changes in this study. Similar ranges of deviation were observed under room-temperature and cold-storage conditions. This finding contrasts with previous reports that temperature can significantly influence polymer behavior and dimensional stability [8,11,25]. One possible explanation is that the temperature range evaluated here was not sufficient to induce measurable differences in polymer relaxation or shrinkage. In addition, the relatively short observation period may have limited the detection of temperature-related effects. Such effects could become more pronounced over longer durations.

Several limitations should be considered when interpreting these results. First, all scans were performed with a clinical intraoral scanner rather than a laboratory-grade scanner. This may introduce additional variability. However, it also enhances clinical relevance by more closely simulating real-world conditions. Second, the observation period was limited to 28 days. This may not capture long-term dimensional changes, which have been reported over several weeks or months. Finally, only two storage conditions were evaluated—room temperature and cold storage. Extreme temperature scenarios, which can induce more pronounced changes, were not included. Future studies should consider extended follow-up periods, a wider range of environmental conditions including humidity control, and additional materials.

## 5. Conclusions

Three-dimensional printed models exhibited material- and region-dependent dimensional changes. Over the short-term, one-month study period, these changes were not affected by storage temperature. Although gypsum models initially demonstrated greater distortion, they remained more stable over time. Conversely, the resin models exhibited lower initial distortion but greater temporal changes, with significant differences observed among the materials. Nevertheless, the observed dimensional changes were likely clinically negligible for most prosthodontic applications. Long-term follow-up, preferably over one year, is required to determine whether more substantial dimensional changes develop over time.

## Figures and Tables

**Table 1:** Regional trueness values (mm) of the conventional gypsum control group (G) and the printed model groups: Shining3D Dental Model Resin DM12 (SH), BEGO Varseo Wax Model Gray (B), and Keystone KeyModel Ultra Resin (K). Measurements are presented across the anterior region (A), left and right middle regions (LM and RM), left and right posterior regions (LP and RP), and the total value (Total) at day 0 and day 28 under room-temperature storage (RT) and cold storage (C). The total value represents the overall mean trueness calculated from all measured regions. Lower values indicate superior trueness. Upper letters “A” indicates statistically significant differences between time points. Lower letters “a” indicates no statistically significant difference.

	Total	LP	LM	A	RM	RP
C-B-0	0.115 ^A^	0.118 ^A^	0.118 ^a^	0.119 ^a^	0.101 ^a^	0.117 ^A^
C-B-28	0.128 ^A^	0.130 ^A^	0.132 ^a^	0.133 ^a^	0.114 ^a^	0.130 ^A^
C-G-0	0.124 ^a^	0.097 ^a^	0.098 ^a^	0.155 ^a^	0.104 ^a^	0.160 ^a^
C-G-28	0.127 ^a^	0.103 ^a^	0.103 ^a^	0.155 ^a^	0.110 ^a^	0.160 ^a^
C-K-0	0.063 ^A^	0.071 ^A^	0.067 ^A^	0.061 ^a^	0.049 ^A^	0.076 ^A^
C-K-28	0.055 ^A^	0.053 ^A^	0.057 ^A^	0.064 ^a^	0.043 ^A^	0.052 ^A^
C-SH-0	0.070 ^A^	0.058 ^a^	0.082 ^A^	0.084 ^a^	0.055 ^A^	0.059 ^a^
C-SH-28	0.064 ^A^	0.052 ^a^	0.076 ^A^	0.079 ^a^	0.050 ^A^	0.053 ^a^
RT-B-0	0.108 ^A^	0.106 ^a^	0.117 ^a^	0.108 ^a^	0.099 ^a^	0.108 ^A^
RT-B-28	0.116 ^A^	0.123 ^a^	0.123 ^a^	0.112 ^a^	0.104 ^a^	0.125 ^A^
RT-G-0	0.148 ^a^	0.143 ^a^	0.121 ^a^	0.133 ^a^	0.146 ^a^	0.227 ^a^
RT-G-28	0.152 ^a^	0.150 ^a^	0.123 ^a^	0.141 ^a^	0.145 ^a^	0.228 ^a^
RT-K-0	0.069 ^A^	0.080 ^A^	0.080 ^A^	0.063 ^a^	0.054 ^A^	0.076 ^A^
RT-K-28	0.056 ^A^	0.054 ^A^	0.063 ^A^	0.061 ^a^	0.043 ^A^	0.057 ^A^
RT-SH-0	0.068 ^a^	0.058 ^A^	0.080 ^a^	0.079 ^a^	0.054 ^a^	0.056 ^A^
RT-SH-28	0.068 ^a^	0.066 ^A^	0.075 ^a^	0.077 ^a^	0.052 ^a^	0.064 ^A^

## Data Availability

The data supporting the findings of this study are available from the corresponding author upon reasonable request.

## Abbreviations

The following abbreviations are used in this manuscript:

DLP	Direct Light Processing
SLA	Stereolithography
LCD	Liquid Crystal Display
STL	Standard Tessellation Language (file format)
IOS	Intraoral Scanner
RMS	Root Mean Square
HLM	Hierarchical Linear Modeling
BHLM	Bayesian Hierarchical Linear Model
SH	Shining (Shining3D resin group)
B	Bego (Varseo Wax Model Gray group)
K	Keystone (KeyModel Ultra group)
G	Gypsum group
RP	Right Posterior
LP	Left Posterior
RM	Right Middle
LM	Left Middle
A	Anterior
RT	Room Temperature (Storage conditions)
C	Cold (Storage condition)
Pd	Probability of Direction
